# Monitoring resistance to methomyl and synergism in the non-target *Musca domestica* from cotton fields of Punjab and Sindh provinces, Pakistan

**DOI:** 10.1038/s41598-023-34331-4

**Published:** 2023-05-01

**Authors:** Hafiz Azhar Ali Khan

**Affiliations:** Department of Entomology, University of the Punjab, Lahore, Pakistan

**Keywords:** Environmental impact, Entomology

## Abstract

Insecticides are an integral part of most of the cropping systems worldwide; however, these usually exert negative impact on the environment and non-target insects as well. Non-target insects are prone to develop resistance to insecticides due to prolonged and repeated lethal and sublethal exposures. *Musca domestica* is a common non-target, pollinator and nectar feeder species in cotton ecosystem, besides its status as a public health pest in human habitations. In the present work, resistance to methomyl, one of the major insecticides used for cotton pest management, was assessed in 20 *M. domestica* strains from the major cotton producing areas of the Punjab and Sindh provinces of Pakistan. The results revealed that toxicity values of methomyl for Punjabi and Sindhi strains ranged from 28.07 to 136.16 µg fly^−1^ and 29.32 to 136.87 µg fly^−1^, respectively. Among Punjabi strains, D.G. Khan, Lodhran, Bahawalpur, Toba Tek Singh, Bahawalnagar, Rajanpur and Jhang strains exhibited very high levels of resistance (RR > 100) to methomyl; Bhakkar, Kasur, Vehari, Layyah, Muzaffargarh and R.Y. Khan showed high resistance (RR = 51–100 fold), while the Mianwali strain showed a moderate level of resistance to methomyl (RR = 36.45 fold). In case of Sindhi strains, very high levels of resistance (> 100 fold) were reported for Sukkar and Sanghar strains, high levels of resistance (RR 51–100 fold) for Khairpur, Jamshoro and Ghotki, and moderate resistance to methomyl (38.08 fold) in the Dadu strain. There was a significant synergism of methomyl toxicity in all field strains when methomyl bioassayed along with piperonyl butoxide (PBO) and *S,S,S*-tributylphosphorotrithioate (DEF) providing clues of metabolic-based mechanisms of resistance to methomyl. In conclusion, insecticides used in crop farming can cause resistance development in non-target *M. domestica*. It is necessary to adopt the pest management activities that are safe for the environment and non-target insect species.

## Introduction

Conventional crop farming systems still rely heavily on the use of agrochemicals to manage pests and diseases and to ensure a high yield of crops to feed a continuously increasing human population^1^. The use of insecticides, one of the agrochemicals, to manage target insect pests is among the major agricultural management practices that may affect negatively non-target species (e.g., insects, nematodes, earthworms, molluscs) and other connected environmental resources^2,3^. Therefore, it is the need of the hour to protect the environment and non-target insect species while managing target insect pests^1^.

Non-target species in or around insecticides-treated areas receive insecticides via residual, topical or through dietary exposures that result in various toxicity symptoms^4^. Acute and chronic toxicity of insecticides in non-target species usually depends on several factors such as the type of insecticides, the frequency of application, the level of uptake and metabolization, non-target species in question, its age, life stage, tolerance and adaptation to insecticides^5–7^. In addition, the time of exposure to a particular insecticide is also important in risk assessment studies that results in the accumulation of insecticide residues in the exposed species and produce a delayed toxic effect with the passage of time^8,9^. As a result of acute and chronic exposure to insecticides, exposed species exhibited arrested growth and lengthened-development period^10^ or may evolve resistance to insecticides due to continued selection pressure^11^.

Insecticide resistance is a genetic change in response to continued selection pressure by insecticides that ultimately results in impaired chemical control in the field^12^. The development of resistance to insecticide in non-target species could be more alarming in situations where the species is a major pest in another situation^13^. For instance, the presence of human- or animal-diseases vectors such as mosquitoes and flies would be non-target species in cropping areas, and if they developed resistance to insecticides, they become difficult to control when expended to nearby human populated areas^14–16^.

Pakistan is among the major cotton (*Gossypium hirsutum* L.) producing countries of the world, with around 2.79 million hectares cultivated area annually^17^. Cotton is mainly cultivated in the two major provinces of Pakistan i.e., Punjab and Sindh. Management of insect pests of cotton is important to ensure a high yield of the crop. Therefore, farmers heavily rely on the use of insecticides as a major insect pest management tool in both of the provinces^18,19^. Methomyl is among the most widely and commonly used insecticides for the management of a number of insect pests of cotton such as bollworms, armyworm, aphids, mealy bug, dusky bug, jassids and whiteflies^20,21^. It is a broad-spectrum insecticide and belongs to the carbamate class of insecticides. In Pakistan, it is being used usually in the form of spray applications for the last four decades, due to which resistance have been reported in different target insect species^21–26^. It is believed that the use of insecticides on crops also results in lethal and sublethal exposure to non-target insect species around farming areas as well^1,27^. Previously, resistance to mathomyl in the non-target mosquito *Aedes albopictus* (Skuse) was reported from the cotton fields of Punjab, Pakistan^28^. The house fly, *Musca domestica* Linnaeus, is a public health pest and one of the most common non-target insect species in cotton cultivated areas^29^. *Musca domestica* has been reported as one of pollinator and nectar feeder species of cotton crop^29–33^. In this way, it is expected that *M. domestica* get residues of methomyl after its application during flight, pollination and/or nectar feeding activities into the cotton fields, and develop resistance to methomyl after prolonged and repeated exposures.

Therefore, the present study was planned to check the hypothesis that the use of methomyl in cotton cultivation has caused resistance development in the non-target *M. domestica* collected from the cotton fields of major cotton producing areas of the Punjab and Sindh provinces of Pakistan.

## Materials and methods

### *Musca domestica* strains

**Figure 1:**
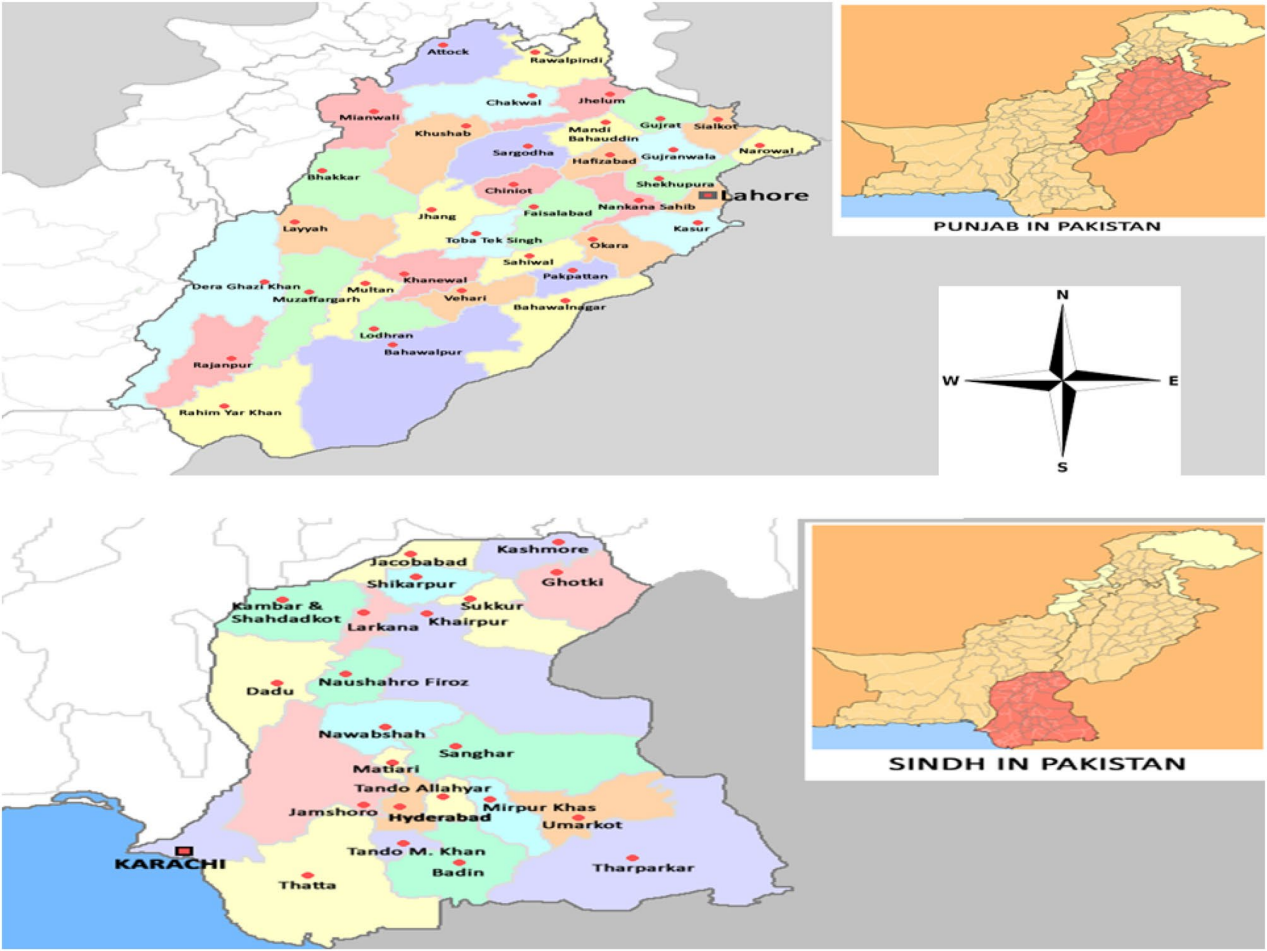
Collection sites of *Musca domestica* from the cotton fields of Punjab and Sindh provinces of Pakistan (*Source* Wikimedia commons).

Twenty field strains of *M. domestica* were collected from major cotton producing localities of the Punjab and Sindh provinces of Pakistan (Fig. 1). Localities from the Punjab province included: Bhakkar (31.6082° N, 71.0854° E), Kasur (31.1179° N, 74.4408° E), Jhang (31.2781° N, 72.3317° E), Rajanpur (29.1044° N, 70.3301° E), Lodhran (29.5467° N, 71.6276° E), Bahawalpur (29.3544° N, 71.6911° E), Bahawalnagar (30.0025° N, 73.2412° E), Dera Ghazi (D.G.) Khan (30.0489° N, 70.6455° E), Rahim Yar (R.Y.) Khan (28.4212° N, 70.2989° E), Mianwali (32.5839° N, 71.5370° E), Toba Tek Singh (30.9709° N, 72.4826° E), Muzaffargarh (30.0736° N, 71.1805° E), Layyah (30.9693° N, 70.9428° E) and Vehari (30.0442° N, 72.3441° E)^34^. Localities from the Sindh province included: Sukkur (27.7244° N, 68.8228° E), Khairpur (27.5256° N, 68.7551° E), Jamshoro (25.4304° N, 68.2809° E), Ghotki (28.0271° N, 69.3235° E), Sanghar (26.0436° N, 68.9480° E) and Dadu (26.7341° N, 67.7795° E)^34^. Cotton fields for *M. domestica* collection in the above localities were chosen based on history of methomyl use for the management of insect pests of cotton (personal communication with regional farmers and agriculture extension workers). An insecticide susceptible reference strain (Lab-susceptible) of *M. domestica*^35,36^ was used in bioassays for the estimation of resistance to methomyl in field strains. Field strains were collected at the adult stage and brought to the laboratory of entomology, University of the Punjab, Lahore (31.5204° N, 74.3587° E)^34^. All strains were reared under the laboratory conditions (12:12 h dark/light photoperiod, 26 ± 2 °C, and 65 ± 5% relative humidity) following a well-established methodology using a sugar–milk-based diet^37,38^. Flies were reared in mesh cages, and pupae of specific date/time duration of a preceding generation were separated and kept in a new/empty cage for starting the subsequent generation. Adults from pupae usually emerged in 4–5 days; in this way flies of required age could easily be collected for bioassays. The first generation (F1) of field-collected strains was used for bioassays.

### Bioassays and data analyses

Technical-grade methomyl (> 95% purity; Chem Service Inc, West Chester PA) was used for resistance detection bioassays in field strains of *M. domestica*. Topical bioassay method was used to apply methomyl doses on *M. domestica* as stated earlier in author's previous reports ^15,39^:

“Briefly, 0.5 μL of insecticide in acetone solution was applied by using a micropipette (0.1–2 µL, Acura ® manual 825, Socorex, Switzerland) on thoracic notum of 3–5-day-old female *M. domestica*. *M. domestica* were exposed to a range of methomyl doses that caused > 0 and < 100% mortality, and each bioassay was consisted of 20 M*. domestica* per dose. In the control treatment, flies were treated with acetone alone. Treated flies were kept in plastic jars (250 mL) provided with a cotton dental wick soaked with 20% sugar solution. All the bioassays were conducted at 26 ± 2 °C, 60 ± 5% RH, 12:12 (L/D) photoperiod, and replicated three times on different days. Mortality counts were made 48-h post-treatment and the data were analyzed by Probit analysis (Finney 1971) to determine median lethal doses (LD_50s_) of insecticides tested. Resistance ratios (RRs) were calculated by dividing LD_50_ values of different field strains to those obtained with the Lab-susceptible reference strain, and categorized as high resistance (RR = 51–100 fold), moderate resistance (RR = 21–50 fold), low resistance (RR = 11–20 fold), very low resistance (RR = 2–10 fold) and no resistance (RR = 1)”^15,39^.

For synergism bioassays, *M. domestica* were exposed topically to enzyme inhibitors: piperonyl butoxide (PBO) and *S,S,S*-tributylphosphorotrithioate (DEF) (Chem Service Inc, West Chester PA), with the maximum sublethal dose of 10 µg fly^−1^, one hour before the insecticide treatment^40,41^. Treated *M. domestica* were then exposed to methomyl doses as stated above. Synergism ratio (SR) was calculated by dividing the LD_50_ value of a particular strain to methomyl alone by the LD_50_ value of methomyl of the corresponding strain along with PBO or DEF. The SR value was considered significantly different if its 95% fiducial limit (FL) did not include “1” on the basis of the ratio test^42^.

## Results

Field strains of *M. domestica* collected from different localities of the Punjab and Sindh provinces exhibited variable toxicity and resistance levels to methomyl compared with the Lab-susceptible strain (Table 1). The Lab-susceptible strain was the most susceptible one among all strains of *M. domestica* with the LD_50_ value 0.77 µg fly^−1^. Toxicity values of methomyl for Punjabi strains ranged from 28.07 to 136.16 µg fly^−1^. Amomg Punjabi strains of *M. domestica*, the Mianwali strain exhibited the lowest LD_50_ value (28.07 µg fly^−1^) for methomyl followed by R.Y. Khan (42.65 µg fly^−1^), Muzaffargarh (47.10 µg fly^−1^), Layyah (49.14 µg fly^−1^), Vehari (49.71 µg fly^−1^), Kasur (62.88 µg fly^−1^), Bhakkar (72.22 µg fly^−1^), Jhang (80.16 µg fly^−1^), Rajanpur (87.13 µg fly^−1^), Bahawalnagar (116.01 µg fly^−1^), Toba Tek Singh (117.78 µg fly^−1^), Bahawalpur (116.01 µg fly^−1^), Lodhran (124.38 µg fly^−1^) and D.G. Khan (136.16 µg fly^−1^) strains. In the case of Sindhi strains of *M. domestica*, the highest toxicity of methomyl was recorded in the Dadu strain (29.32 µg fly^−1^) followed by Ghotki (44.92 µg fly^−1^), Jamshoro (50.68 µg fly^−1^), Khairpur (74.64 µg fly^−1^), Sanghar (80.41 µg fly^−1^) and Sukkur (136.87 µg fly^−1^) strains (Table 1).

**Table 1:** Toxicity and resistance levels of *Musca domestica* strains to methomyl.

Strain	n*	LD_50_ (µg fly^−1^)**	Fit of probit line				RR (95% FL)***
			Slope (SE)	χ^2^	df	*P*	
Lab-susceptible	480	0.77 (0.66–0.90)	3.41 (0.43)	3.87	5	0.57	–
Bhakkar	480	72.22 (59.87–92.84)	1.49 (0.14)	1.90	5	0.86	93.79 (70.70–125.24)
Kasur	480	62.88 (52.14–76.83)	1.82 (0.16)	2.12	5	0.83	81.66 (63.67–105.45)
Jhang	420	80.16 (64.56–102.34)	1.58 (0.25)	1.64	4	0.80	104.10 (78.95–138.22)
Rajanpur	420	87.13 (69.83–108.70)	1.53 (0.16)	3.11	4	0.54	113.16 (86.40–149.20)
Lodhran	420	124.38 (100.51–156.50)	1.57 (0.17)	3.10	4	0.54	161.53 (123.33–212.97)
Bahawalpur	420	121.83 (91.38–165.33)	2.09 (0.28)	5.33	4	0.26	158.22 (125.00–201.61)
Bahawalnagar	420	116.01 (82.27–168.10)	1.90 (0.17)	6.59	4	0.16	150.66 (117.95–193.73)
D.G. Khan	420	136.16 (90.73–222.43)	1.60 (0.31)	7.20	4	0.13	176.83 (134.89–233.35)
R.Y. Khan	420	42.65 (31.17–57.87)	2.19 (0.48)	6.21	4	0.18	55.39 (43.98–70.22)
Mianwali	420	28.07 (18.78–40.77)	2.40 (0.21)	9.57	4	0.05	36.45 (29.12–45.96)
Toba Tek Singh	420	117.78 (91.32–154.43)	2.17 (0.19)	4.45	4	0.35	152.96 (121.22–194.30)
Muzaffargarh	420	47.10 (36.48–60.87)	2.13 (0.18)	4.25	4	0.37	61.17 (48.47–77.70)
Layyah	420	49.14 (42.44–56.92)	2.76 (0.23)	1.65	4	0.80	63.82 (51.50–79.61)
Vehari	480	49.71 (41.49–60.00)	1.89 (0.15)	1.54	5	0.82	64.56 (50.69–82.78)
Sukkur	480	136.87 (88.39–234.50)	2.01 (0.17)	7.23	5	0.20	177.75 (139.47–228.05)
Khairpur	480	74.64 (55.58–100.68)	1.89 (0.35)	7.42	5	0.19	96.94 (76.23–124.09)
Jamshoro	480	50.68 (42.05–60.91)	1.84 (0.15)	4.24	5	0.52	65.82 (51.64–84.44)
Ghotki	480	44.92 (36.48–54.82)	1.63 (0.13)	1.78	5	0.88	58.34 (36.47–54.82)
Sanghar	480	80.41 (67.60–96.01)	2.01 (0.46)	3.91	5	0.56	104.43 (82.54–133.00)
Dadu	480	29.32 (26.07–39.36)	1.94 (0.17)	7.68	5	0.17	38.08 (29.84–48.92)

*Number tested.

**Median lethal dose determined after 48 h exposure to methomyl.

***Resistance ratio.

Resistance ratio (RR) values ranged from 36.45 to 176.83 fold for Punjabi strains, and 38.08 to 177.75 fold for Sindhi strains, compared with the Lab-susceptible strain of *M. domestica* (Table 1). Among Punjabi strains, D.G. Khan, Lodhran, Bahawalpur, Toba Tek Singh, Bahawalnagar, Rajanpur and Jhang strains exhibited very high levels of resistance (RR > 100) to methomyl; Bhakkar, Kasur, Vehari, Layyah, Muzaffargarh and R.Y. Khan showed high resistance (RR = 51–100 fold), while the Mianwali strain showed a moderate level of resistance to methomyl (RR = 36.45 fold). In case of Sindhi strains, very high levels of resistance (> 100 fold) were reported for Sukkar and Sanghar strains, high levels of resistance (RR 51–100 fold) for Khairpur, Jamshoro and Ghotki, and moderate resistance to methomyl (38.08 fold) in the Dadu strain (Table 1).

Except the Lab-susceptible strain, toxicity of methomyl in all field strains increased significantly, based on non-overlapping 95% FL of LD_50_ values and significant synergism ratios (SR), when bioassayed along with either PBO or DEF (Table 2). For instance, the LD_50_ value of the Bhakkar strain reduced (toxicity increased) from 72.22 to 24.33 and 26.74 µg fly^−1^ when bioassayed in the presence of PBO and DEF, respectively. The LD_50_ value of the Kasur strain reduced from 62.88 to 33.05 and 32.21 µg fly^−1^ when bioassayed in the presence of PBO and DEF, respectively. The LD_50_ value reduced from 80.16 to 33.15 and 24.47 µg fly^−1^ for the Jhang strain; from 87.13 to 20.40 and 29.17 µg fly^−1^ for the Rajanpur strain; from 124.38 to 77.00 and 67.61 µg fly^−1^, when bioassayed in the presence of PBO and DEF, respectively. A similar trend of increased toxicity was observed with the rest of the Punjabi and Sindhi strains of *M. domestica*. Synergism ratios were significant in all the cases of field strains based on 95% FL of SR values did not include 01 (Table 2).

**Table 2:** Synergism of methomyl toxicity by the enzyme inhibitors in different strains of *Musca domestica.*

Strain	Methomyl	n	LD_50_ (µg fly^−1^)	Fit of probit line				SR (95% FL)*
				Slope (SE)	χ^2^	df	*P*	
Lab-susceptible	+ PBO	480	0.72 (0.54–0.95)	2.29 (0.19)	5.61	5	0.35	1.07 (0.86–1.37)
	+ DEF	480	0.78 (0.66–0.94)	2.07 (0.18)	3.88	5	0.57	0.99 (0.78–1.26)
Bhakkar	+ PBO	420	24.33 (17.03–38.53)	1.76 (0.21)	3.34	4	0.50	2.97 (2.15–4.11)
	+ DEF	420	26.74 (16.25–57.87)	2.06 (0.23)	7.85	4	0.10	2.70 (1.98–3.68)
Kasur	+ PBO	480	33.05 (28.02–39.29)	2.25 (0.34)	2.70	5	0.75	1.90 (1.47–2.46)
	+ DEF	480	32.21 (27.74–37.58)	2.66 (0.23)	3.17	5	0.67	1.95 (1.52–2.50)
Jhang	+ PBO	420	33.15 (27.70–39.74)	2.12 (0.31)	2.11	4	0.72	2.42 (1.81–3.24)
	+ DEF	420	24.47 (13.94–40.21)	2.32 (0.24)	8.10	4	0.09	3.28 (2.46–4.36)
Rajanpur	+ PBO	420	20.40 (16.55–24.55)	2.04 (0.22)	0.12	4	0.99	4.27 (3.18–5.74)
	+ DEF	420	29.17 (23.98–35.30)	1.95 (0.21)	0.89	4	0.93	2.99 (2.23–4.01)
Lodhran	+ PBO	420	77.00 (60.40–95.29)	1.44 (0.19)	1.75	4	0.78	1.62 (1.16–2.26)
	+ DEF	420	67.61 (49.38–92.75)	2.57 (0.25)	4.15	4	0.39	1.84 (1.40–2.41)
Bahawalpur	+ PBO	420	53.88 (32.84–83.94)	2.25 (0.19)	5.64	4	0.23	2.26 (1.77–2.89)
	+ DEF	420	36.84 (28.74–47.20)	3.06 (0.46)	5.97	4	0.20	3.31 (2.64–4.15)
Bahawalnagar	+ PBO	420	75.07 (62.30–79.98)	1.95 (0.17)	1.26	4	0.87	1.54 (1.19–2.01)
	+ DEF	420	66.67 (49.23–79.13)	2.21 (0.19)	5.75	4	0.22	1.74 (1.35–2.25)
D.G. Khan	+ PBO	420	18.14 (13.55–24.30)	2.67 (0.23)	7.04	4	0.13	7.51 (5.74–9.82)
	+ DEF	420	29.17 (22.83–36.74)	2.44 (0.22)	4.22	4	0.38	4.66 (3.55–6.14)
R.Y. Khan	+ PBO	420	30.13 (22.35–41.48)	2.55 (0.32)	7.31	4	0.12	1.42 (1.12–1.78)
	+ DEF	420	26.57 (16.48–45.34)	2.25 (0.19)	8.70	4	0.07	1.60 (1.26–2.04)
Mianwali	+ PBO	420	18.27 (15.08–22.02)	1.88 (0.17)	3.49	4	0.48	1.54 (1.20–1.97)
	+ DEF	420	16.39 (13.80–19.36)	2.22 (0.20)	2.65	4	0.62	1.71 (1.35–2.17)
Toba Tek Singh	+ PBO	420	17.54 (10.69–28.34)	2.27 (0.41)	8.04	4	0.09	6.71 (5.28–8.54)
	+ DEF	420	24.71 (16.69–37.56)	2.72 (0.23)	9.03	4	0.06	4.77 (3.79–5.99)
Muzaffargarh	+ PBO	420	6.67 (4.90–8.88)	2.50 (0.22)	6.47	4	0.17	7.06 (5.58–8.94)
	+ DEF	420	8.81 (6.90–11.22)	2.56 (0.22)	4.78	4	0.31	5.35 (4.24–6.74)
Layyah	+ PBO	420	28.62 (21.95–37.87)	2.33 (0.14)	5.26	4	0.26	1.72 (1.38–2.14)
	+ DEF	420	23.31 (14.80–37.75)	2.54 (0.31)	8.95	4	0.06	2.11 (1.70–2.61)
Vehari	+ PBO	480	15.50 (11.95–20.17)	1.95 (0.16)	6.10	5	0.30	3.21 (2.48–4.15)
	+ DEF	420	13.48 (11.44–15.94)	2.26 (0.19)	3.45	4	0.49	3.65 (2.87–4.74)
Sukkur	+ PBO	480	42.72 (34.05–53.30)	2.17 (0.17)	5.10	5	0.40	3.20 (2.49–4.12)
	+ DEF	420	40.99 (32.64–51.39)	2.49 (0.21)	4.11	4	0.39	3.34 (2.62–4.26)
Khairpur	+ PBO	480	40.89 (31.36–51.25)	2.10 (0.18)	4.46	5	0.49	1.83 (1.42–2.35)
	+ DEF	480	34.84 (21.26–52.56)	1.99 (0.18)	8.78	5	0.12	2.14 (1.66–2.77)
Jamshoro	+ PBO	480	17.27 (14.57–20.34)	2.24 (0.19)	1.26	5	0.94	2.93 (2.28–3.77)
	+ DEF	480	23.64 (1.68–2.74)	2.41 (0.37)	2.98	5	0.70	2.14 (1.68–2.74)
Ghotki	+ PBO	480	19.11 (13.98–26.44)	2.27 (0.17)	8.11	5	0.15	2.35 (1.81–3.06)
	+ DEF	480	14.43 (12.37–16.85)	2.46 (0.20)	2.51	5	0.77	3.11 (2.41–4.03)
Sanghar	+ PBO	480	16.31 (10.97–22.97)	1.52 (0.14)	7.51	5	0.19	4.93 (3.71–6.56)
	+ DEF	480	22.14 (16.48–29.43)	1.94 (0.16)	7.26	5	0.20	3.63 (2.82–4.68)
Dadu	+ PBO	420	20.29 (13.43–24.61)	1.97 (0.18)	9.04	4	0.06	1.44 (1.11–1.88)
	+ DEF	420	13.07 (6.98–17.33)	2.26 (0.21)	7.37	4	0.12	2.24 (1.74–2.89)

*Synergism ratio.

## Discussion

It is a matter of great concern that the use of pesticides in agriculture often poses negative impact on non-target organisms, including insect species^43,44^. Based on the data of last few years, it has been observed that the use of insecticides in cropping systems resulted in the occurrence of resistance in the target and non-target insect species in Pakistan, which shows that this phenomenon is extremely widespread^1,15^. The present work could be considered as a continuation of our efforts to explore side-effects of insecticidal usage in agriculture on non-target insect species, providing additional data of the impact of methomyl on *M. domestica* from localities not already explored. The data clearly indicates the occurrence of field-evolved resistance to methomyl in field strains of *M. domestica* in comparison to the Lab-susceptible strain. According to Valles et al.^45^, an insect strain should be assumed resistant to a particular insecticide if it shows more than tenfold RR value in comparison to the susceptible or reference strain. The data of the present study revealed that all of the field strains were resistant to methomyl and exhibited more than tenfold RR values in comparison to the Lab-susceptible strain.

The susceptibility of reference strains of *M. domestica* to methomyl varies in different reports, depending upon strain origin, rearing conditions, and/or bioassay methods. While this does not undermine such studies, it is valuable to refer literature estimates as a rough means of comparison^46^. In the present study, the LD_50_ value of methomyl for the Lab-susceptible strain (0.77 µg fly^−1^), is greater than UCR (0.58 µg fly^−1^)^46^, WHO (0.10 µg fly^−1^)^47^ and Cooper (0.07 µg fly^−1^)^48^ strains. Resistance ratio values for both Punjabi and Sindhi strains ranged from moderate to high levels, compared with the Lab-susceptible strain of *M. domestica*. Among Punjabi strains, D.G. Khan, Lodhran, Bahawalpur, Toba Tek Singh, Bahawalnagar, Rajanpur and Jhang strains exhibited very high levels of resistance (RR > 100) to methomyl; Bhakkar, Kasur, Vehari, Layyah, Muzaffargarh and R.Y. Khan showed high resistance (RR = 51–100 fold), while the Mianwali strain showed a moderate level of resistance to methomyl (RR = 36.45 fold). In case of Sindhi strains, very high levels of resistance (> 100 fold) were reported for Sukkar and Sanghar strains, high levels of resistance (RR 51–100 fold) for Khairpur, Jamshoro and Ghotki, and moderate resistance to methomyl (38.08 fold) in the Dadu strain. Resistance to methomyl could be due to the fact that field strains were collected from the cotton fields where methomyl was being used as one of the major insecticides to manage different insect pests such as bollworms, armyworm, aphids, mealy bug, dusky bug, jassids and whiteflies^20,49^. It is assumed that variation in resistance levels or toxicity in different strains might be due to the differences in origin of strains, climatic factors of collection sites and/or history of insecticide exposure. Variations in toxicity to insecticides due to these reasons have also been documented for different insect pests^50–56^.

Variable levels of resistance to methomyl in *M. domestica* have been reported from different countries in the past^46,57–59^. Previously, we also have reported low levels of resistance to methomyl in *M. domestica* strains collected from dairy farms in different localities, other than the ones in the present work, of Punjab, Pakistan^37^. Methomyl was used to target/manage *M. domestica* in dairy farms. However, in the present study, *M. domestica* strains were collected from the cotton fields where these are non-target species. Insecticidal usage in crops, besides controlling target pests, usually results in the lethal and sublethal exposures to non-target species that ultimately make these species resistant to insecticides with the passage of time^10,11^. Recently, resistance development has been reported in *M. domestica* and *Aedes albopictus* due to non-targeted exposure to insecticides used in rice farming^1,15^. Methomyl formulation has been registered in the form of emulsifiable concentrate (EC) and applied as sprays to manage different insect pests of cotton in Pakistan. Sprays of insecticides contaminate plant parts, soil, water and the surrounding air for a certain period of time^11,15^. It is believed that *M. domestica* get direct and/or indirect exposure to methomyl sprays during their routine life activities and developed resistance to methomyl as evidenced by the data of the present study.

Resistance to methomyl could be due to the activation of metabolic enzymes such as microsomal oxidases, esterases, etc., which can be initially checked by the use of enzyme inhibitors along with insecticides in bioassays^51,60^. Synergism of methomyl by PBO and DEF in *Helicoverpa armigera* (Hübner)^60^, *M. domestica*^61^ and *Oxycarenus hyalinipennis* Costa^26^ inferred that resistance may be attributable to microsomal oxidase and esterase detoxification. In the present work, synergism of methomyl with PBO and DEF in all the field strains was observed suggesting the possibility of metabolic mechanism of resistance. More in vitro studies are needed to further confirm the role of metabolic resistance mechanism in field strains of *M. domestica*.

## Conclusion

The finding that non-target *M. domestica* has evolved resistance to methomyl used for the management of insect pests of cotton is troubling evidence of the side-effects of insecticidal usage in crop farming. The development of insecticide resistance in non-target species as a result of insecticide application against the targeted species usually lead to the outbreak of former species^11^. *M. domestica* is one of the major medical and veterinary pests and the development of resistance to insecticides may promote its outbreak coupled with an increased incidence of fly-borne diseases. Therefore, it is important to perform risk assessment studies in order to determine side-effects of a particular insecticide on non-target species before and after its approval for use in cropping systems.

## Data Availability

The data presented in this study are available in article.
